# Human longevity is characterised by high thyroid stimulating hormone secretion without altered energy metabolism

**DOI:** 10.1038/srep11525

**Published:** 2015-06-19

**Authors:** S. W. Jansen, A. A. Akintola, F. Roelfsema, E. van der Spoel, C. M. Cobbaert, B. E. Ballieux, P. Egri, Z. Kvarta-Papp, B. Gereben, C. Fekete, P. E. Slagboom, J. van der Grond, B. A. Demeneix, H. Pijl, R. G. J. Westendorp, D. van Heemst

**Affiliations:** 1 Department of Gerontology and Geriatrics, Leiden University Medical Centre, Leiden, The Netherlands; 2 Department of Medicine, Section Endocrinology, Leiden University Medical Centre, Leiden, The Netherlands; 3 Department of Clinical Chemistry and Laboratory Medicine, Leiden University Medical Centre, Leiden, The Netherlands; 4 Department of Endocrine Neurobiology, Institute of Experimental Medicine, Hungarian Academy of Sciences, Budapest, Hungary; 5 Semmelweis University, János Szentágothai PhD School of Neurosciences, Budapest, H-1085 Hungary; 6 Department of Medicine, Division of Endocrinology, Diabetes and Metabolism, Tupper Research Institute, Tufts Medical Centre, Boston, MA, USA; 7 Section of Molecular Epidemiology, Department of Medical Statistics, Leiden University Medical Centre, Leiden, The Netherlands; 8 Department of Radiology, Leiden University Medical Centre, Leiden, The Netherlands; 9 UMR 7221 CNRS / MNHN Evolution des Régulations Endocriniennes, Département Régulations, Développement et Diversité Moléculaire, Muséum National d’Histoire Naturelle, Paris, France; 10 Department of Public Health, University of Copenhagen, Denmark

## Abstract

Few studies have included subjects with the propensity to reach old age in good health, with the aim to disentangle mechanisms contributing to staying healthier for longer. The hypothalamic-pituitary-thyroid (HPT) axis maintains circulating levels of thyroid stimulating hormone (TSH) and thyroid hormone (TH) in an inverse relationship. Greater longevity has been associated with higher TSH and lower TH levels, but mechanisms underlying TSH/TH differences and longevity remain unknown. The HPT axis plays a pivotal role in growth, development and energy metabolism. We report that offspring of nonagenarians with at least one nonagenarian sibling have increased TSH secretion but similar bioactivity of TSH and similar TH levels compared to controls. Healthy offspring and spousal controls had similar resting metabolic rate and core body temperature. We propose that pleiotropic effects of the HPT axis may favour longevity without altering energy metabolism.

Worldwide, the population of elderly is rapidly growing, with numbers expected to further increase in the coming decades. Although epidemiological studies have discovered specific lifestyle and genetic risk factors for cardiovascular disease, dementia and cancer, age is unequivocally the major common risk factor^1^. With the expansion of the ageing population, the prevalence of all major age related diseases will increase, including cardiovascular disease, diabetes mellitus (type 2) and dementia. Most studies have included diseased subjects with the aim to identify risk factors for specific diseases^2^. Only few studies have included subjects with a propensity to reach old age in good health, with the aim to disentangle mechanisms contributing to healthy human longevity and protection from disease. The Leiden Longevity Study (LLS) comprises nonagenarians with at least one nonagenarian sibling, their offspring and the offspring’s partners^3^. Compared to their partners, offspring from nonagenarian siblings have a lower mortality rate, a lower prevalence of diabetes and cardiovascular disease^3^ and are therefore well suited for studying the mechanisms underlying healthy human longevity.

Numerous theories of ageing link energy metabolism to the ageing process. The “rate of living theory” postulates that the positive correlation between lifespan and size implicates species differences in resting metabolic rate^4^. The mechanistically linked “free radical theory of ageing” proposes that free radicals generated as by-products of oxidative metabolism underpin the negative correlation between lifespan and resting metabolic rate^5^. Other theories propose ageing to involve precocious depletion of functional stem cell reserves^6^ which might be retarded by slowing tissue turnover rates. One physiological integrator that influences metabolism, growth, development and tissue turnover is thyroid signalling. In previous studies, we found that when nonagenarians were stratified for their propensity to reach advanced age, those with the lowest family mortality history score had the highest TSH levels and slightly lower levels of free T4 (fT4) and free T3 (fT3)^7^. When offspring were compared to their partners, fT4 levels were similar, whereas TSH was higher and fT3 levels were slightly lower^8^,^9^. Moreover studies in the oldest old from the general population also link increased levels of TSH with reduced old age mortality^10^.

In this study, we are the first that make use of frequent blood sampling in relation with familial longevity to study TSH secretion and TH levels over 24 hours, because TSH and fT3 were shown to have circadian rhythms^11^. In addition, we investigated whether such differences in TSH and/or TH concur with differences in energy metabolism, a physiological process that has been associated with longevity in model organisms and is known to be responsive to TSH/TH action.

## Results

### Baseline characteristics

Between 2002 and 2006, 421 families with at least two long-lived Caucasian siblings were recruited in the Leiden Longevity Study, without any selection on health. Males had to be aged 89 years or above and females 91 years or above^3^,^12^. For the current study (Switchbox) we included 61 offspring and 51 partners in which we measured energy metabolism (the full study sample) and 20 offspring and 18 partners (subgroup) in which we sampled blood continuously over 24 hours.

The baseline characteristics of both the full study sample as well as the subgroup are presented in Table 1. In the full study sample as well as in the subgroup, the groups of offspring from long-lived families and partners were of similar age, sex and BMI. Participants were selected on the basis of the age of their parents. Consequently, in both groups, mothers of offspring were significantly older (*P* < 0.001), and in the full study sample the age of the offspring’s fathers was significantly higher as well (*P* < 0.001). In line with previous findings^3^, offspring had less cardiovascular disease.

### TSH and thyroid hormones

**Figure 1:**
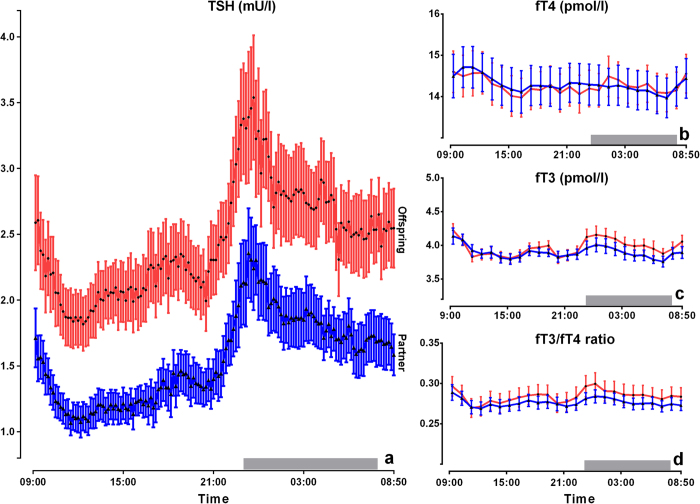
Twenty-four hour profiles of TSH, fT4, fT3 and fT3/fT4 ratio in offspring from long-lived families and partners. In all the panels, data points represent means with standard error of the mean. The red lines depict 20 offspring and the blue lines depict 18 partners. (**a**) Ten minutes measurements of TSH. Hourly measurements of (**b**) fT4 (**c**) fT3 (**d**) fT3/fT4 ratio. The grey bars represent lights-off periods (from 23:00-08:00).

Offspring from long-lived siblings had on average 0.8 mU/l higher serum concentrations of TSH at all time points over a 24-hour period (Fig. 1a). We calculated the area under the curve (AUC) to estimate hormone production over the 24 -hour period, during the day period (9.00 h–23.00 h) and during the night (24.00 h-06.00 h). The mean (95% CI) TSH AUC over the 24- hour period was significantly (*P* = 0.009) higher in the offspring (56.1 (45.7–6.5 mU/l)) compared to that of their partners (35.3 (24.4–46.3) mU/l) (Supplementary Table 1). Moreover, the mean (95% CI) TSH AUC was significantly higher during the day (*P* = 0.006) as well as during the night (*P* = 0.025) in the offspring compared to the partners thereof. The 24-hour profiles of fT4 (Fig. 1b) and fT3 (Fig. 1c) and the areas under the curves over the 24-hour period, during the day and night (Supplementary Table 1) did not differ between groups. We performed deconvolution analyses to quantify total TSH secretion over 24 hours on the basis of the TSH concentration profiles^13^. Geometric mean (95% CI) total TSH secretion over 24 hours was significantly (*P* = 0.007) higher in the offspring (55.0 (43.9–68.9) mU/l) compared to partners (34.4 (27.1–43.7) mU/l).

To ensure that the subgroup represented the full study sample, we replicated the TSH and TH measurements in a fasted single late morning sample for the 112 subjects from the full study sample (Table 2). Again, we found significantly increased (*P* = 0.01) geometric mean (95% CI) serum concentrations of TSH in offspring (2.1 (1.8–2.3) mU/l) compared to partners (1.6 (1.4–1.9) mU/l), but no difference in TH serum concentrations.

### Regularity of consecutive serum TSH concentration measurements over 24 hours

ApEn was used as a regularity statistic to quantify the regularity or orderliness of consecutive serum TSH concentration measurements over 24 hours, with a higher ApEn indicating a greater irregularity^14^,^15^. Geometric mean (95% CI) ApEn of TSH was similar between offspring and partners ((1.26 (1.13–1.41) versus (1.15 (1.02–1.29) *P* = 0.22)).

### Relationship between circulating TSH and fT4

In the human population, the HPT axis maintains circulating TSH and thyroid hormone levels in a physiological inverse relationship. We calculated the fT4xTSH product and the fT4/TSH ratio to further characterize the relationship between circulating TSH and fT4 in offspring and partners. In both the full study sample and in the subgroup we found a significantly higher fT4xTSH product in offspring compared to partners (Table 2). In the full study sample and subgroup, fT4/TSH ratio was significantly lower in the offspring compared to partners (Table 2). In addition, in the subgroup, offspring had a significantly lower (*P* = 0.01) geometric mean (95% CI) AUC fT4/total TSH production ratio (6.1 (4.8–7.8)) compared to partners (9.7 (7.5–12.7)).

### Estimates of peripheral deiodination

A possible mechanism that could underlie the offspring’s increased TSH secretion is enhanced TH turnover due to increased uptake in tissues or hormone clearance. Amongst others, one process that could contribute to peripheral TH turnover is deiodination^16^. To estimate conversion of fT4 into fT3, we calculated the fT3/fT4 ratio in both the full study sample and the subgroup (Table 2). In both the full study sample and in the subgroup no significant differences in fT3/fT4 ratio were found between offspring and partners thereof. Moreover, we calculated the AUCfT3/AUCfT4 ratio in the subgroup only, as a more precise measure of deiodination over the whole 24 -hour period. However, no significant difference was observed in mean (95% CI) AUCfT3/AUCfT4 ratio between offspring and partners (6.8 (6.3–7.2) versus (6.6 (6.1–7.0), *P* = 0.56)). Deiodinase 1 and deiodinase 2 are responsible for the generation of T3 from T4, whereas deiodinase 3 is the major inactivating enzyme leading to an increase of reverse T3 (rT3). The ratio between T3 and rT3 (T3/rT3 ratio) is therefore regarded as a more sensitive marker of peripheral thyroid hormone metabolism^17^,^18^,^19^. No significant difference was observed in T3/rT3 ratio between offspring and partners in both the full study sample as well as in the subgroup (Table 2). Also after exclusion of 1 female partner, who had a rT3 measurement above the reference range, no difference was found between offspring and partners in the mean (95% CI) T3/rT3 ratio (6.8 (6.3–7.2) versus 6.5 (5.9–7.0) *P* = 0.39).

### TSH bioactivity

**Figure 2:**
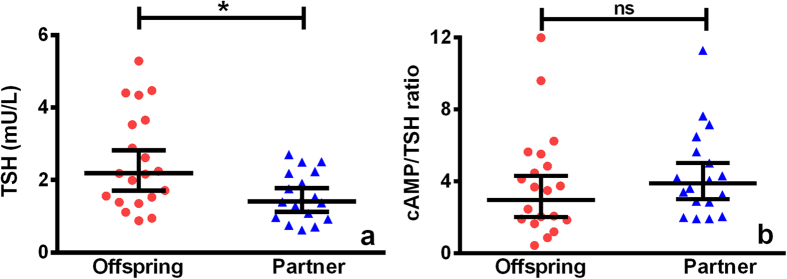
*In vitro* TSH bioactivity in offspring from long-lived siblings and partners. Red circles represent 20 offspring, blue triangles represent 18 partners. Solid lines represent (**a**) geometric mean with 95% CI of TSH levels (mU/l) (**b**) geometric mean with 95% CI of cAMP/TSH ratio. *P < 0.05; ns: not significant.

We determined TSH bioactivity *in vitro* to assess whether the offspring’s increased TSH secretion was a compensatory mechanism for reduced TSH bioactivity. We first assessed if the TSH levels in the sample in which we wanted to measure TSH bioactivity were higher in the offspring (Fig. 2a). Again, we found a significant (*P* < 0.02) higher mean (95% CI) TSH level in offspring (2.2 (1.7–2.8) mU/l) compared to partners thereof (1.4 (1.1–1.8) mU/l). To determine whether the bioactivity of TSH molecules was different between the groups, the total amount of cAMP produced was adjusted for sample TSH concentrations, by calculating the cAMP/TSH ratio which was similar for offspring and partners (Fig. 2b).

### Metabolism

**Figure 3:**
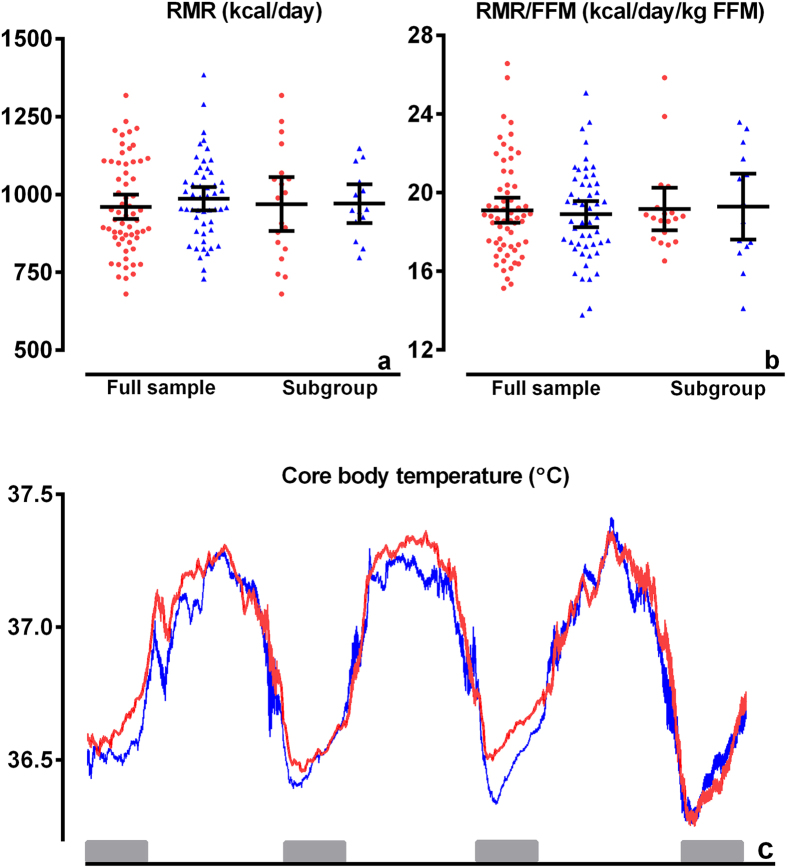
Parameters of energy metabolism in offspring from long-lived siblings and partners. Red circles represent offspring (n = 61 for full study sample; n = 20 for subgroup) and blue triangles represent partners (n = 51 for full study sample; n = 18 for subgroup). (**a**) Mean (95% CI) resting metabolic rate (kcal/day). (**b**) Mean (95% CI) resting metabolic rate per kg fat free mass (**c**) Mean (SEM) core body temperature per 5 minutes over 3 days in full study sample offspring (red line) and partners (blue line). Grey blocks are the night periods (24.00h–07.00 h). RMR: resting metabolic rate; FFM: fat free mass. *P ≤ 0.05; **P ≤ 0.01; ns: not significant.

Mean (95% CI) resting metabolic rate in offspring and partners did not differ in the full study sample (960 (924–997) kcal/day versus (987 (947–1026) kcal/day, *P* = 0.34)) nor in the subgroup (Fig. 3a). Adjustments for age and sex did not materially change the results. Moreover, resting metabolic rate per kg fat free mass (FFM) did not differ between offspring and partners in both groups (Fig. 3b). There was no difference in core body temperature over the 3 day period between offspring and partners in the full study sample (Fig. 3c) or in the subgroup.

## Discussion

In this study, we investigated if human longevity is associated with differences in TSH and/or TH and whether these concur with differences in energy metabolism because this process is responsive to TSH/TH action and has been associated with longevity in model organisms. The main finding of this study is that familial longevity is characterized by higher TSH secretion, in the absence of differences in TH levels and metabolism.

The association of higher TSH with familial longevity is in line with our earlier observations in nonagenarians from the Leiden Longevity Study^7^ and their offspring^9^ as well as with earlier observations in Ashkenazi centenarians and their offspring^20^. In line with the data described in this article, higher TSH in Ashkenazi centenarians and their offspring was not associated with lower concentrations of circulating thyroid hormone levels^20^. In contrast, previously we did find slightly lower levels of thyroid hormones in offspring from long-lived siblings^8^ and we also found that nonagenarians from families with the lowest family mortality history score had relatively lower levels of thyroid hormones^7^. However, these previous studies were performed using non-fasted single blood samples randomly taken over the day and there were no^7^ or less stringent^8^ selection criteria on health status of the participants, which can both influence TH levels. In addition, the observed differences between offspring and partners with respect to the thyroid hormones were very small^8^. In the current study, to reduce confounding by health status, we used more strict inclusion criteria (as described in the methods) and to reduce confounding by sampling time, all comparisons of serum TSH and thyroid hormone measurements between groups were standardized for sampling time.

The observation that despite increased levels of TSH, levels of TH were similar between groups aligns with the observation that energy metabolism was not different between groups. Metabolic rate is inversely associated with longevity in different animals and metabolism plays a central role in several ageing theories. In turn, administration of TH is well known to increase metabolic rate in diverse species, including humans. Recent studies in animals have also implicated central mechanisms in the effects of T3 on increased metabolism^21^. We did not find differences in resting metabolic rate or core body temperature between the offspring and the partners in the full study sample nor in the subgroup. This is remarkable, since many ageing theories and longevity models in animals are related to changes in energy metabolism. These findings align with the observation that despite increased levels of TSH, levels of TH were similar between groups. Also the fT3/fT4 ratio, as a proxy for conversion of fT4 into fT3 was comparable between offspring and partners both in the full study sample and in the subgroup. Moreover in the subgroup the AUCfT3/AUCfT4 ratio over the 24 -hour period did not significantly differ between offspring and partners.

Several possible mechanisms can contribute to the observed increase in TSH in the offspring group, including (i) reduced bioactivity of circulating TSH, (ii) diminished sensitivity of thyrotrophs to negative feedback by thyroid hormones, (iii) diminished responsiveness of the thyroid gland to TSH, (iv) enhanced thyroid hormone turnover in peripheral tissues and (v) enhanced clearance of thyroid hormones from the circulation.

The bioactivity of circulating TSH is known to differ depending on the degree of glycosylation and sialylation. A lower TSH bioactivity in the offspring would require higher concentrations of circulating TSH to maintain circulating thyroid hormone levels. In our study sample, TSH bioactivity, as reflected by the cAMP/TSH ratio in an *in vitro* activity assay, was similar in the offspring and partners.

To explore the possibility that the offspring might have higher TSH because of diminished sensitivity of thyrotrophs to negative feedback by thyroid hormones, we calculated the fT4xTSH product and found that it was higher in the offspring group. Previously, the fT4xTSH product has been used to quantitate the sensitivity of the thyroptrophs to feedback regulation by thyroid hormone and the degree of inherited thyroid hormone resistance^22^. Although all subjects with inherited thyroid hormone resistance had higher serum concentrations of fT4 for corresponding TSH concentrations, the degree of thyroid hormone resistance differed depending on the type of mutation, which was reflected by the fT4xTSH product^22^. Thus, a higher thyrotroph T4 resistance index in the offspring might be indicative of reduced sensitivity of the thyrotrophs to feedback regulation by thyroid hormone. However, there are two observations that might argue against this interpretation. First, one would expect the increased TSH secretion to result in an increase in fT4 concentrations. However, in contrast to subjects with thyroid hormone resistance^22^, circulating thyroid hormone levels were not higher in the offspring. Second, the ApEn of TSH secretion was not significantly different in the subgroup between offspring and partners. ApEn of TSH was used as a regularity statistic to quantify the regularity or orderliness of TSH secretion, with a higher ApEn indicating a greater irregularity. Mathematical models and feedback experiments have established that pattern orderliness monitors feedback and/or feedforward interactions within different hypothalamic-pituitary target-organ systems with high sensitivity and specificity^14^. With regard to TSH secretion, previous studies have shown that the ApEn of TSH secretion is greatly increased in patients with severe hypothyroidism, but not in subjects with subclinical hypothyroidism or in controls^15^. Thus, in severe hypothyroid patients, the failure of the thyroid gland to produce sufficient amounts of thyroid hormone will lead to loss of thyroid hormone mediated feedback on TRH and TSH secretion, and this was reflected by an increase in the irregularity of TSH secretion (higher ApEn of TSH secretion). In contrast, in patients with subclinical hypothyroidism, thyroid hormone levels are within the normal range, and the ApEn of TSH secretion was unchanged, reflecting intact thyroid hormone mediated feedback on TRH and TSH secretion^23^.

An alternative interpretation for the increased fT4xTSH product in offspring is that it reflects higher serum concentrations of TSH for corresponding fT4 concentrations. This finding may thus hint more towards reduced responsiveness of the thyroid gland to TSH, driving production of TSH to maintain sufficient circulating fT4 and fT3 levels. We further explored the possibility that TSH secretion could compensate for reduced responsiveness of the thyroid gland by calculating the fT4/TSH ratio in the full study sample and the AUC fT4/total TSH production ratio. We found that these ratio’s were significantly lower in the offspring lending support to the possibility that their thyroid gland is likely to be less responsive to TSH than the thyroid gland of their partners.

A fourth possible mechanism underlying increased TSH secretion is enhanced TH turnover. The fT3/fT4 ratio, a proxy for conversion of fT4 into fT3 did not differ between offspring and partners. However, while circulating levels of fT4 and fT3 are kept within narrow ranges, TH levels exhibit much wider variation within target tissues due to intense and highly regulated control of tissue- specific TH action by deiodinases, transporters and transcriptional co-regulators. Thus, we cannot exclude the possibility that TSH levels are increased in familial longevity due to increased TH turnover in target tissues. Although we did not have data on deiodinase activity in specific target tissues, circulating T3/rT3 ratio was shown to exhibit a strong and positive correlation with liver deiodinase 1 activity^17^. In our study, no differences were found between offspring and partners in the T3/rT3 ratio. Taken together, enhanced TH turnover by increased peripheral turnover of TH is less likely to be an underlying mechanism of the increased TSH secretion.

It should be noted that the sample size of the current study is relatively small and that differences that did not reach statistical significance in our study sample might be clinically significant. The families included in the Leiden Longevity Study are genetically heterogeneous, and thus different mechanisms may be at play in different families and/or in different individuals.

A fifth possible mechanism underlying increased TSH secretion is that the offspring may have an enhanced rate of thyroid hormone clearance from the circulation, which would trigger the pituitary to secrete more TSH, stimulating the production of thyroid hormones to ensure appropriate circulating TH concentrations. Unfortunately, we did not have data on thyroid hormone clearance by the liver and kidneys to evaluate this possibility.

Thus, our data cannot discriminate which mechanism is responsible for the altered thyroid status in familial longevity and what the physiological consequences are of the observed increased TSH secretion. Future studies should aim to disentangle the causes and consequences of the altered thyroid status by TSH and TH challenges.

Moreover, this study did not investigate other processes that might be influenced by differences in TSH and/or TH and that might be relevant for longevity. One such key process is tissue turnover. In adult mammalian tissues, damaged and worn-out mature cells are continuously being replaced during normal tissue homeostasis and in response to stresses and injury, a process that is critically dependent on the differentiation of self-renewing, tissue-specific stem cells. Various theories propose that ageing implicates either depletion or failed differentiation of stem cells^6^. The TSH receptor (TSHR), a G-protein coupled hormone receptor, plays an important role in growth and differentiation of thyroid follicular cells. However, TSHR expression is not limited to the thyroid. TSHR is also expressed on bone cells^24^,^25^ and other cells, including adipocytes, hepatocytes, skeletal muscle cells, neuronal cells, astrocytes and mesenchymal stem cells^26^,^27^,^28^. In mesenchymal stem cells TSH induces gene expression patterns that have been implicated in functions related to stem cell fate, including self-renewal, differentiation and maintenance^26^. TSH is also involved in cellular differentiation in other cells with effects depending on the stage of differentiation, e.g. TSH stimulated early differentiation of preadipocytes but inhibited their proliferation and terminal differentiation^29^. However, because of the physiological inverse relationship between TSH and thyroid hormones, and the critical role of thyroid hormone in development, the extrathyroidal effects of TSH in the skeleton and other tissues remain controversial^24^.

A strength of our study is that we frequently measured hormone levels over a 24- hour period. Therefore, we were able to standardize comparison of hormone levels between groups for clock time and were able to explore differences in total TSH secretion using deconvolution analysis. Another strength is that we simultaneously measured several physiological parameters known to be affected by thyroid hormones. Since we included offspring enriched for longevity and compared them with their partners, we had a matched control group. One of the weaknesses of the study is that not all offspring are enriched for longevity causing us to underestimate actual effects. Moreover, the invasive nature and the high costs of hormone rhythm studies make it impossible to perform such studies using a large sample size and relatively small but clinically significant differences between groups may not be detected.

The principal finding of this study is that familial longevity is characterized by higher TSH secretion, in the absence of differences in TH concentration and in whole body energy metabolism. Taken together, these observations suggest that pleiotropic effects of the HPT axis protect long-lived families. Further in depth mechanistic studies should focus on disentangling the underlying mechanisms.

## Methods

### Participants

Between 2002 and 2006, 421 families with at least two long-lived Caucasian siblings, 1671 of their offspring and 744 of the offspring’s partners were recruited in the Leiden Longevity Study, without any selection on health. Males had to be aged 89 years or above and females 91 years or above^3^,^12^. For the current study (Switchbox), between March 2012 and July 2013, 135 offspring and partners from the LLS were measured at the study centre of the Leiden University Medical Centre. Inclusion criteria included being middle-aged (55–77 years) and having a stable body mass index (BMI) between 19 kg/m^2^ and 33 kg/m^2^. Participants were excluded if their fasting plasma glucose was above 7 mmol/l, if they had any significant chronic, renal, hepatic or endocrine disease, or if they used any medication known to influence lipolysis, thyroid function, glucose metabolism, GH/IGF-1 secretion or any other hormonal axis. Moreover, participants were excluded if they had a recent trans meridian flight, smoking addiction, use of more than 20 units of alcohol per week and extreme diet therapies. Other exclusion criteria specific for the subgroup only, were difficulties to insert and maintain an intravenous catheter, anemia (hemoglobin < 7.1 mmol/l), and blood donation within the last two months. Based on information obtained via telephone questioning, controls with a nonagenarian parent who had one or more nonagenarian siblings were also excluded. All women in this study were postmenopausal. The Switchbox protocol was approved by the Medical Ethical Committee of the Leiden University Medical Centre and was performed according to the Helsinki declaration. All participants gave written informed consent for participation.

Of the 135 subjects included, we excluded from analysis 17 subjects due to incomplete indirect calorimetry data and 6 subjects due to incomplete core body temperature data (Supplementary Fig. 1). Thus, data from 112 subjects (61 offspring, 51 partners) were available for analysis (and are referred to as full study sample). Complete series of 24 -hour blood samples were obtained for a subgroup of 38 participants (20 offspring, 18 partners).

### Continuous blood sampling

Participants were sampled in the same research room and received standardized feeding at three fixed times during the day (between 09.00 h–10.00 h, 12.00 h–13.00 h and 18.00–19.00 h), each consisting of 600 kcal Nutridrink (Nutricia Advanced Medical Nutrition, Zoetermeer, The Netherlands). No naps were allowed during the day and lights were turned off between 23.00 h to 08.00 h. A catheter was placed in a vein of the forearm of the non-dominant hand. Every 10 minutes, 1.2 ml of blood was collected in K_3_-EDTA tube and 2 ml in a serum-separator (SST)-tube. In total, 460.8 ml of blood was withdrawn from each participant.

### Processing of the samples

After blood withdrawal, the K3-EDTA tubes were immediately placed on ice before centrifugation. Serum tubes were kept at room temperature and centrifuged when the samples were clotted, usually between 30–60 minutes. Samples were centrifuged at 3520 RPM at 4 °C for 10 minutes. The EDTA plasma and serum samples were stored in two aliquots of 500 μl during the rest of the sampling at −20 °C. After the sampling they were transferred to a −80 °C freezer until analysis.

### Chemical analyses

All measurements were performed at the Department of Clinical Chemistry and Laboratory Medicine, Leiden University Medical Centre, The Netherlands. All laboratory measurements were performed with fully automated equipment and diagnostics from Roche Diagnostics (Almere, The Netherlands). Aspartate Aminotransferase (AST) (Catalog number 11876848216), Alanine Aminotransferase (ALT) (Catalog number 11876805216) and creatinine (Catalog numbers R1 11875566216, R2 11875582216) were measured from a fasted late morning serum sample using the Modular P800 clinical chemistry analyser. TSH (Catalog number 11731459122), fT4 (Catalog number 6437281190), fT3 (Catalog number 13051986190), and T3 (Catalog number 11731360122) were measured in serum by ElectroChemoLuminescence ImmunoAssay (ECLIA) using a Modular E170 Immunoanalyzer. The TSH method has been standardized against the 2nd IRP WHO Reference Standard 80/558. For each participant, all samples from one time series were measured with the same lot number in the same batch. For fT4 and fT3, all samples were measured in one batch. For TSH, measurements were made in 10 batches. For each couple, offspring and partner were measured with the same lot number and preferentially also in the same batch. For this study, the precision and quality of all assayed analytes met or surpassed the level of desirable quality specifications^30^. The coefficients of variation (CVs) for AST, ALT, creatinine , and fT3 were all below their advised levels of 6.0% for AST, 12.2% for ALT, 2.2% for creatinine, and 4.4% for T3. For TSH, fT4 and fT3 Randox controls (catalog numbers IA 3109 and IA 3111) were used to determine the CVs. For TSH, the CV ranged in our study between 1.41–4.16, which was well below the desired CV of 9.9%. For fT4, the CV range in our study was 2.41–3.49 and for fT3 2.16–2.91, both well below the upper CV limits for desired precision of these assays (fT4 ≤ 3.8 and fT3 ≤  4.0). In our laboratory, the reference values for TSH were 0.3–4.8  mU/l, for fT4 10–24  pmol/l, for fT3 3–8 pmol/l and for T3 1.1–3.1 nmol/l.

### rT3 measurements

All rT3 measurements were measured in the same batch for both the full study sample as well as for the subgroup. Serum rT3 levels were measured with in-house radioimmunoassays by the Laboratory of Endocrinology and Radiochemistry of the Academic Medical Centre in Amsterdam^31^ with an intra-assay variation between 4–5% and an inter-assay variation between 5–9% and a detection limit of 0.03 nmol/l. The reference range of rT3 was 0.11–0.44 nmol/l.

### Measurements of metabolism

On study day 1, between 12.00 h and 13.00 h, participants had a 30 minutes indirect calorimetry session using a ventilated hood system (Care Fusion Canopy Jaeger Oxycon Pro, Houten, The Netherlands) after 14 hours of fasting. Participants were kept under standardised conditions, lying awake and emotionally undisturbed, completely at rest and comfortably supine on a bed, their head under a transparent ventilated canopy, in a thermally neutral environment. From the VO_2_ and VCO_2_ measurements, resting metabolic rate (RMR) was calculated using the formula 3.91 VO_2_ + 1.10 VCO_2_ – 1.93N^32^.

Body composition was measured using a Bioelectrical Impedance Analysis meter at a fixed frequency of 50kHz (Bodystat® 1500 Ltd, Isle of Man, British Isles)^33^. Participants wore the Equivital monitor (Equivital EQ02 SEM, Hidalgo, UK) for the measurements of core body temperature (CBT) for five consecutive days (Supplementary Fig. 2).

### Data processing of the core body temperature

In order to assess core body temperature, each participant swallowed one Core Body Temperature Capsule (Capsule REF 500-0100-02Respironics Inc., Murrysville, PA, USA) at each of three consecutive days, during dinner time (Supplementary Fig. 2). The capsule measured the core body temperature at a frequency of 250 ms and was connected with the Equivital device by radio emission, with a maximum range of one meter. The acceptable core body temperature sensing range of the capsule is from 32 °C to 42 °C, according to factory settings. After a variable number of hours or days the core temperature capsule was discarded with the faeces.

Means per 5 minutes were calculated based on measurements every 15 seconds, temperature measurements ≤35 °C or ≥41 °C were excluded, and the first 5 hours after ingestion of the pill were removed to limit the influence of intake of food on the core body temperature measurements before passage through the stomach^34^.

### Single measurements of thyroid hormones

Fasted blood samples were taken in the late morning for the measurement of TSH, fT4 and fT3.

### TSH bioactivity

For the *in vitro* measurement of TSH bioactivity we used the 09.10 h K_3_-EDTA-samples of the 38 participants who were frequently sampled over 24 hours. We measured the biological activity of TSH *in vitro* according to our established protocol based on the recommendation of the American Thyroid Association Guide by measuring intracellular cAMP production of cultured Chinese hamster ovary cells stably transfected with the human TSH receptor (kindly provided by Dr. AC Bianco, Chicago, USA)^35^,^36^. The levels of cAMP were measured using a double-antibody radioimmunoassay kit (adenosine 3’5’cyclic monophosphate, PerkinElmer®, Massachusetts, USA).

### Deconvolution analysis

The 24-hour TSH secretion was analyzed using a recently validated deconvolution method^13^.

### Approximate entropy (ApEn)

ApEn of TSH was used as a regularity statistic to quantify the regularity or orderliness of consecutive serum TSH concentration measurements over 24 hours. Mathematical models and feedback experiments establish that pattern orderliness monitors feedback and/or feed-forward interactions within an interlinked axis with high sensitivity and specificity, both greater than 90%^37^. Reduced pattern regularity typifies hormone secretion in puberty and ageing, during diminished negative feedback or fixed exogenous stimulation, and by autonomous neuroendocrine tumours^38^.

### Statistical analysis

Descriptive statistics were used to summarise the characteristics of both study groups. Chi square test and t-test were used to describe differences between offspring and partners regarding sex, age, age of the parents, body composition, medical history, medication, creatinine clearance, liver function tests, smoking and alcohol use. To calculate differences in secretion between offspring and partner for the different thyroid status parameters, areas under the curves were calculated according to the trapezoid method using SigmaPlot for Windows Version 11.0 (Systat Software, GmbH, Erkrath, Germany). The areas under the curves were calculated over the 24 hours, during the day from 09.00 h to 23.00 h and during the night from 24.00 h to 06.00 h. Linear regression was used to compare total TSH secretion, levels of TH, T3, rT3, measurements of AUCs, levels of cAMP/TSH ratio, AUCfT4/total TSH secretion, fT4/TSH ratio, fT4xTSH product, fT3/fT4 ratio, T3/rT3 ratio, AUC fT3/AUC fT4 ratio and parameters of the energy metabolism between offspring and partners adjusted for age and sex. When not normally distributed, parameters were log transformed for analysis, and data are presented as geometric mean with 95% confidence interval. Because ApEn of TSH was still not normally distributed after log transformation, a non-parametric test was used. For all above mentioned analyses the Statistical Package for the Social Sciences program for Windows, version 20 (SPSS, Chicago, IL) was used. Graphs were made using GraphPad Prism version 5 (GraphPad, San Diego, CA).

## Additional Information

**How to cite this article**: Jansen, S. W. *et al.* Human longevity is characterised by high thyroid stimulating hormone secretion without altered energy metabolism. *Sci. Rep.* **5**, 11525; doi: 10.1038/srep11525 (2015).

## Supplementary Material

Supplementary Information

## Figures and Tables

**Table 1:** Baseline characteristics of full the study sample and subgroup.

	**Full study sample**			**Subgroup**		
**Offspring n** **=** **61**	**Partner n** **=** **51**	**P-value**	**Offspring n** **=** **20**	**Partner n** **=** **18**	**P-value**	
Demographics						
Male n (%)	28 (45.9)	27 (52.9)	0.46	10 (50.0)	10 (55.6)	0.73
Age (years)	65.9 (6.4)	65.9 (6.1)	0.95	65.6 (5.4)	64.6 (4.9)	0.52
Age mother (years)†	89.7 (10.3)	77.5 (15.3)	**<0.001**	92.4 (7.9)	78.6 (13.9)	**0.001**
Age father (years)†	86.0 (15.1)	72.6 (11.5)	**<0.001**	82.5 (18.9)	76.8 (9.2)	0.25
BMI (kg/m^2^)	25.6 (3.6)	26.5 (4.3)	0.22	25.4 (4.0)	25.5 (3.9)	0.91
Fat mass (kg)*	23.9 (6.6)	26.4 (9.8)	0.13	23.5 (7.1)	23.7 (7.8)	0.93
Fat free mass (kg)*	51.4 (11.8)	53.1 (10.4)	0.42	51.3 (12.0)	52.5 (11.4)	0.75
Medical history						
Cardiovascular disease n (%)	2 (3.2)	7(13.7)	**0.04**	0 (0)	1 (5.5)	0.29
Malignancies n (%)	7 (11.5)	2 (3.9)	0.14	3 (15.0)	0 (0)	0.09
Osteoporosis/arthritis n (%)	5 (8.2)	5 (9.8)	0.77	1 (5.0)	2 (11.1)	0.49
Medication						
Statins n (%)	4 (6.6)	9 (17.6)	0.07	0 (0)	1 (5.6)	0.29
Anti-hypertensive n (%)	11 (18.0)	17 (33.3)	0.06	3 (15.0)	2 (11.1)	0.72
Laboratory results						
Creatinine clearance (ml/min)	87.0 (21.2)	91.6 (27.1)	0.32	89.9 (18.7)	94.7 (22.0)	0.47
Aspartate Aminotransferase (U/l)	26.0 (8.9)	27.3 (13.1)	0.51	25.9 (6.2)	24.4 (6.9)	0.50
Alanine Aminotransferase (U/l)	25.0 (22.4)	24.4 (9.1)	0.86	23.4 (5.2)	23.1 (7.1)	0.88
Lifestyle						
Smoking current n (%)	1 (1.6)	1 (2.0)	0.90	0 (0)	1 (5.6)	0.29
Alcohol >20 units/week n(%)	5 (8.2)	4 (7.8)	0.93	1 (5.0)	2 (11.1)	0.49

Unless indicated otherwise, data are presented as mean (standard deviation).

^*^Data were not available for 1 male partner due to technical problems.

^†^missing data of 3 participants in the full study sample.

**Table 2:** Thyroid status in offspring from long-lived siblings and partners.

	Full study sample*			**Subgroup**		
	**Offspring (n** **=** **61)**	**Partner (n** **=** **51)**	**P-value**	**Offspring (n** **=** **20)**	**Partner (n** **=** **18)**	**P-value**
Hormone levels (rv)						
TSH (0.3–4.8 mU/l)†	2.1 (1.8–2.3)	1.6 (1.4–1.9)	**0.01**	2.2 (1.7–2.8)	1.4 (1.1–1.8)	**0.02**
fT4 (10–24 pmol/l)	16.1 (15.5–16.7)	16.5 (15.9–17.1)	0.36	14.7 (13.7–15.7)	14.5 (13.5–15.5)	0.78
fT3 (4.7–8.2 pmol/l)	4.7 (4.6–4.8)	4.6 (4.5–4.8)	0.49	4.2 (4.10–4.4)	4.1 (3.9–4.3)	0.36
T3 (1.1–3.1 nmol/l)‡	1.66 (1.59–1.72)	1.62 (1.55–1.68)	0.37	1.50 (1.42–1.57)	1.48 (1.40–1.56)	0.72
rT3 (0.11–0.44 nmol/l)‡	0.26 (0.24–0.28)	0.27 (0.25–0.29)	0.30	0.18 (0.16–0.20)	0.17 (0.15–0.19)	0.57
fT4xTSH product†	33.0 (29.0–37.4)	26.4 (23.0–30.3)	0.02	31.7 (25.0–40.0)	20.4 (15.9–26.2)	0.01
fT4/TSH ratio†	6.7 (5.2–8.6)	10.1 (7.7–13.2)	0.03	7.7 (6.7–8.8)	10.1 (8.7–11.8)	0.01
fT3/fT4 ratio	0.30 (0.29–0.31)	0.29 (0.27–0.30)	0.20	0.30 (0.28–0.32)	0.29 (0.27–0.31)	0.55
T3/rT3 ratio‡	6.77 (6.28–7.25)	6.36 (5.84–6.88)	0.26	8.84 (7.80–9.88)	9.24 (8.13–10.34)	0.60

Unless otherwise indicated data are displayed as means with 95% CI adjusted for age and sex.

^*^data were not available in the full study sample for 2 offspring and 2 partners for analyses of fT4, fT3, TSHxfT4 product, fT4/TSH ratio and fT3/fT4 ratio.

^†^geometric mean with 95% CI.

^‡^data were not available for 4 offspring and 1 partner for analysis due to limited amount of blood. rv: reference values.
