# Supplementary material for: Human longevity is characterised by high thyroid stimulating hormone secretion without altered energy metabolism
Source: Sci Rep. 2015 Jun 19;5:11525. doi: 10.1038/srep11525 (PMC4473605; doi:10.1038/srep11525)
Supplement: Supplementary Information [file srep11525-s1.doc]

**Online-only Supplements for**

**Human longevity is characterized by high thyroid stimulating hormone secretion without altered energy metabolism**

Authors: Steffy W. Jansen, MD^1^†, Abimbola A. Akintola, MD^1^†, Ferdinand Roelfsema, MD, PhD^2^, Evie van der Spoel, BSc^1^, Christa M. Cobbaert, PhD^3^, Bart E. Balieux, PhD ^3^, Peter. Egri, MSc^4,5^, Zsuzsanna Kvarta-Papp, MSc ^4^, Balazs Gereben, PhD ^4^, Csaba Fekete, PhD ^4,6^ , P. Eline Slagboom, PhD ^7^, Jeroen van der Grond, PhD ^8^, Barbara A. Demeneix, PhD ^9^, Hanno Pijl, MD, PhD^2^, Rudi G. J. Westendorp, MD, PhD ^1,10^, Diana van Heemst, PhD 1*

*Correspondence to: D.van_Heemst@lumc.nl

†equal contribution of authors

**Supplementary Figure 1 | Switchbox study sample selection process.**


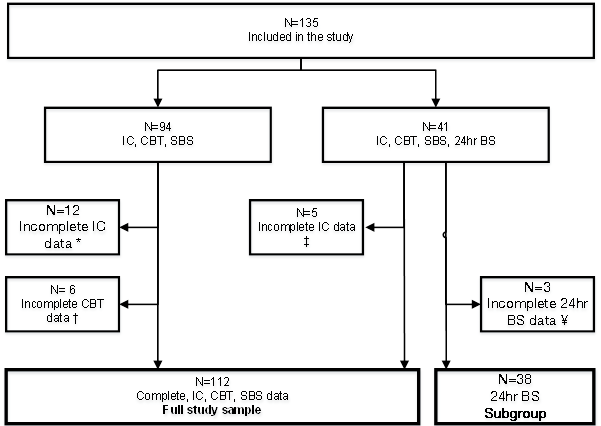


IC: Indirect calorimetry; CBT: Core body temperature measurements; SBS: Single blood sampling; 24hr BS: 24hour blood sampling.

*1 participant excluded due to migraine attack, 2 claustrophobic participants, 9 due to technical failure; †5 due to technical failure, 1 due to inability to wear Equivital belt; ‡1 migraine attack, 2 claustrophobic participants, 2 technical failures; ¥ 3 failed blood samplings due to inability to withdraw blood.

**Supplementary Figure 2 | Study time table for full study sample and the subgroup.**

Supplementary Table 1 | Thyroid status in offspring and partners.

|  | **Offspring n=20** | **Partner n=18** | ***P*-value** |
| --- | --- | --- | --- |
| **AUC TSH (mU/l)** |  |  |  |
| Total AUC | 56.1 (45.7-66.5) | 35.3 (24.4-46.3) | **0.009** |
| AUC during day | 32.6 (26.5-38.8) | 19.6 (13.1-26.1) | **0.006** |
| AUC during night | 16.0 (13.0-19.0) | 10.9 (7.7-14.1) | **0.025** |
| **AUC fT4 (pmol/l)** |  |  |  |
| Total AUC | 339.3 (317.6-361.1) | 338.4 (315.4-361.4) | 0.95 |
| AUC during day | 200.2 (184.6-215.8) | 203.8 (187.3-220.4) | 0.75 |
| AUC during night | 86.9 (80.7-93.2) | 85.1 (78.5-91.6) | 0.68 |
| **AUC fT3 (pmol/l)** |  |  |  |
| Total AUC | 94.3 (91.0-97.7) | 91.8 (88.3-95.3) | 0.30 |
| AUC during day | 54.8 (52.6-56.9) | 54.9 (52.7-57.2) | 0.92 |
| AUC during night | 25.0 (23.6-26.4) | 23.3 (21.8-24.8) | 0.11 |
| **AUC fT3/AUC fT4 TSHxratio** |  |  |  |
| Total AUC | 6.8 (6.3-7.2) | 6.6 (6.1-7.0) | 0.56 |
| AUC during day | 3.9 (3.7-4.2) | 3.8 (3.6-4.1) | 0.59 |
| AUC during night | 2.5 (2.4-2.7) | 2.5 (2.3-2.7) | 0.59 |

Data are displayed as mean with 95% confidence interval adjusted for age and sex. AUC: area under the curve.
